# The Role of Toll-Like Receptors in Skin Host Defense, Psoriasis, and Atopic Dermatitis

**DOI:** 10.1155/2019/1824624

**Published:** 2019-11-14

**Authors:** Lixiang Sun, Wenjie Liu, Ling-juan Zhang

**Affiliations:** ^1^School of Pharmaceutical Sciences, Xiamen University, Xiamen, China; ^2^Department of Dermatology, University of California, San Diego, La Jolla, CA, USA

## Abstract

As the key defense molecules originally identified in Drosophila, Toll-like receptor (TLR) superfamily members play a fundamental role in detecting invading pathogens or damage and initiating the innate immune system of mammalian cells. The skin, the largest organ of the human body, protects the human body by providing a critical physical and immunological active multilayered barrier against invading pathogens and environmental factors. At the first line of defense, the skin is constantly exposed to pathogen-associated molecular patterns (PAMPs) and damage-associated molecular patterns (DAMPs), and TLRs, expressed in a cell type-specific manner by various skin cells, serve as key molecules to recognize PAMPs and DAMPs and to initiate downstream innate immune host responses. While TLR-initiated inflammatory responses are necessary for pathogen clearance and tissue repair, aberrant activation of TLRs will exaggerate T cell-mediated autoimmune activation, leading to unwanted inflammation, and the development of several skin diseases, including psoriasis, atopic dermatitis, systemic lupus erythematosus, diabetic foot ulcers, fibrotic skin diseases, and skin cancers. Together, TLRs are at the interface between innate immunity and adaptive immunity. In this review, we will describe current understanding of the role of TLRs in skin defense and in the pathogenesis of psoriasis and atopic dermatitis, and we will also discuss the development and therapeutic effect of TLR-targeted therapies.

## 1. Introduction

The skin, poised at the interface between the host body and the environment, is constantly exposed to pathogens and environmental insults and therefore has evolved to provide rapid and specific immune responses to these stimuli. Precise and situation-specific innate immune responses of skin cells to insults lead to rapid induction of host defense molecules including antimicrobial peptides (AMPs) and proinflammatory cytokines that shapes the adaptive immune responses, leading to immediate as well as long-term protection against pathogens or physical dangers.

Pattern recognition receptors (PRRs) are the vast array of germline-encoded surveillance receptors responsible for recognizing pathogens, activating the innate immune system, and priming antigen-specific adaptive immunity [1]. Upon infection or injury, pathogen-associated molecular patterns (PAMPs) released by a pathogen or damage-associated molecular patterns (DAMPs) by damaged cells are taken up by affected cells to activate membrane and/or cytosolic PRRs. PAMPs or DAMPs, such as pathogenic or host nucleic acid (DNA or RNA), protein, lipid, or lipoprotein, can be detected by unique PRR and initiate differential downstream signaling cascades, leading to situation-specific host immune responses after bacterial, viral, and parasitic infection and skin injury [2, 3].

Mammals have several highly conserved and distinct classes of PPRs including Toll-like receptors (TLRs), RIG-I-like receptors (RLRs), Nod-like receptors (NLRs), AIM2-like receptors (ALRs), C-type lectin, and intracellular DNA sensors such as cGAS-STING. Among different classes of PRRs, TLRs were the first to be characterized and are the most extensively studied innate immune receptors in both vertebrates and invertebrates [4]. Mammalian TLRs were first identified based on their sequence homology with the Drosophila Toll gene, which was originally discovered by Dr. Jules Hoffmann as the crucial receptor detecting microorganisms and activating the fly's innate immune defense response against bacterial infection [5, 6]. The discovery of Toll-mediated innate immunity in Drosophila soon led to the discovery of mammalian TLRs by providing evidence that resistance to infection is mediated by inducible antimicrobial genes secondary to activation of the TLR signaling pathway. Dr. Hoffmann was therefore awarded one half of the 2011 Nobel Prize in Physiology or Medicine to acknowledge his contribution to innate immunity.

The unique multilayered structure of the skin enables an effective barrier against a relentless barrage of pathogens and insults. Anatomically, the skin comprises three consecutive layers, including the stratified epidermis, the fibroblast-rich dermis, and the dermal fat (also known as dermal white adipose tissue (dWAT)) [7–9]. Keratinocytes (KCs) are the main epidermal cell type (~95%), and the remaining epidermal cells include Langerhans cells, melanocytes, Merkel cells, and infiltrated immune cells. Dermal fibroblasts (dFBs), the major resident cell types in the dermis, are highly heterogeneous [9, 10]. While dFBs located in the upper (papilla) dermis support epidermal growth and regulate hair cycling, dFBs located in the lower (reticular) dermis have the potential to commit to preadipocytes (pAd) and differentiate to adipocytes, forming the last and deepest barrier of the skin, dWAT [9, 10]. PRR-mediated innate immune activation of these skin resident cells by PAMPs or DAMPs leads to the production of antimicrobial peptides as well as proinflammatory cytokines that recruit and activate myeloid and lymphatic immune cells, such as neutrophils, monocytes, dendritic cells, macrophages, and T lymphocytes. A proper interplay between innate and adaptive immune cells confers immediate and long-term immune protection against pathogens and insults.

While PRR activation is essential for inflammatory responses that initiate skin's host defense against invasive pathogens, overactivation of PRRs often leads to uncontrolled inflammation and the subsequent development of autoimmunity and/or inflammatory skin diseases, such as psoriasis, atopic dermatitis, systemic lupus erythematosus, and diabetes-induced impaired wound healing [11–13]. Here, we will review current literatures on the role of TLRs in host defense and how aberrant activation of TLRs leads to the development of psoriasis and atopic dermatitis and recent advances in therapeutic targeting of TLR to treat these skin diseases.

## 2. TLR Signaling

### 2.1. The Structure of TLRs and Their Cognate Ligands

TLRs are type I transmembrane proteins consisting of three domains including an extracellular domain, a single transmembrane domain, and an intracellular tail [14]. The extracellular domain (ectodomains) contains tandem copies of leucine-rich repeats (LRR) that recognize specific PAMPs or DAMPs as a homo- or heterodimer along with a coreceptor molecule. The intracellular tail of TLRs is homologous to that of IL1 receptor, called the Toll/IL1R domain (TIR), and it is required for downstream signaling transduction. Upon PAMP or DAMP recognition, the TIR domain recruits adaptor proteins, such as MyD88 (myeloid differentiation primary response gene 88) or TRIF (TIR domain-containing adaptor inducing IFN*β*), which initiate signaling cascades that activate NF*κ*B (nuclear factor kappa-light-chain-enhancer of activated B cells), MAPKs, or TBK1 (TANK-binding kinase 1) signaling cascades to regulate the expression of cytokines, chemokines, and antimicrobial peptides that ultimately provide host defense against danger signals [15, 16].

**Figure 1:**
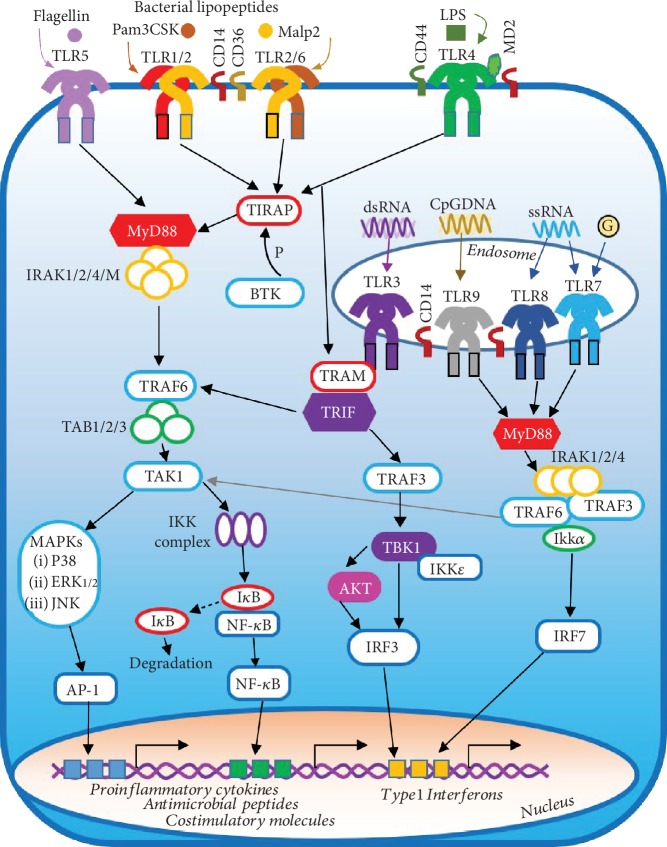
MyD88-dependent and TRIF-dependent TLR signaling pathways. Ligand binding of TLRs by their respective ligands induces dimerization of TLRs and initiates MyD88-dependent or TRIF-dependent signaling cascades. The presence of coreceptors, such as CD14 for TLR2, TLR3, TLR4, TLR7, and TLR9, CD36 for TLR2 and TLR6, and CD44 for TLR4, promotes ligand binding efficiency to TLRs. MD2 is a receptor component associated with TLR4 and enables TLR4 to respond to LPS. Activation of TLR1-TLR2 by the lipopeptide Pam3CSK, TLR2-TLR6 by the lipopeptide Malp2, TLR5 by flagellin, or TLR4 by LPS recruits MyD88 through the adaptor molecule TIRAP. MyD88 then recruits and activates the IRAK complex, which in turn activates TRAF6, which serves as a platform to recruit and activate TAK1 in cooperation with TAB1-3. Once activated, TAK1 activates the IKK-NF*κ*B pathway and the MAPK- (including P38, ERK1/2, and JNK) AP1 pathway. Activated NF*κ*B or AP1 translocates to the nucleus, driving the transcription of genes encoding proinflammatory cytokines, antimicrobial peptides, and costimulatory molecules. Activation of endosomal TLR7 by ssRNA or guanosine, TLR8 by ssRNA, or TLR9 by CpG-DNA not only initiates the MyD88-TRAF6-dependent activation of AP1 and NF*κ*B but also triggers the IRAK-, TRAF6-, TRAF3-, and IKK*α*-dependent activation of IRF7, translocation of which induces the transcription of type1 interferon genes including IFN*α* and IFN*β*. In contrast, activation of TLR3 by dsRNA initiates the TRIF-dependent pathway, whereas TLR4 activation induces both MyD88- and TRIF-dependent pathways. Once recruited to the intracellular domain of TLRs by TRAM, TRIF initiates a TRAF3-dependent activation of the TBK1-type 1 IFN pathway and/or a TRAF6-dependent activation of the TAK1-proinflammatory cytokine pathway. The TRAF3-dependent activation of TBK1 and IKK*ε* and TBK1-mediated activation of AKT result in the coordinate activation of the transcription factor IRF3, which translocates to the nucleus and induces the transcription of type 1 interferon genes upon activation. Pam3CSK4: tripalmitoyl-S-glycero-Cys-(Lys)4; Malp2: macrophage-activating lipopeptide-2; LPS: lipopolysaccharide; dsRNA: double-stranded RNA; ssRNA: single-stranded RNA; CpG: deoxycytidyl-phosphate-deoxyguanosine; MyD88: myeloid differentiation primary response gene 88; TIRAP: TIR domain-containing adaptor protein; TRAM: TRIF-related adaptor molecule; TRIF: TIR domain-containing adaptor inducing IFN*β*; TRAF: TNFR-associated factor; IRAK: IL1R-associated kinase; TAK: transforming growth factor beta-activated kinase 1; TAB: TAK1-binding protein; IKK: inhibitor of nuclear factor kappa-B kinase; NF*κ*B: nuclear factor kappa-light-chain-enhancer of activated B cells; I*κ*B: inhibitor of NF*κ*B; TBK1: TANK binding kinase 1; AMPs: antimicrobial peptides.

To date, 13 mammalian TLRs have been identified and characterized, namely, TLR1 to TLR13, including TLR1-TLR11 in human. Each TLR can interact with specific PAMPs or DAMPs including lipopeptides for TLR1, TLR2, and TLR6, lipopolysaccharide for TLR4, bacterial flagellin for TLR5, dsRNA for TLR3, ssRNA for TLR7 and TLR8, and DNA for TLR9 to initiate various intracellular signaling events triggering innate immune responses (Figure 1). TLRs (TLR1, TLR2, TLR4, TLR5, TLR6, and TLR10) are localized at the cell plasma membrane to sense various cell wall components from gram-positive bacteria or mycoplasma, or protein derivatives from damaged host cells. TLR4, together with its extracellular partner CD14, recognizes lipopolysaccharide (LPS), a cell wall component of gram-negative bacteria. TLR5 recognizes flagellins from either gram-positive or gram-negative bacteria. TLR2 and/or TLR4 can also be activated by endogenous ligands or DAMPs, such as biglycans, hyaluronic acid, heat shock proteins, oxidized lipid, or lipoproteins [17, 18]. In contrast, TLR3, TLR7, TLR8, and TLR9 are localized in the endosome to detect nucleic acids derived from viruses, bacteria, or damaged cells [19]. TLR3 recognizes viral double-stranded RNA (dsRNA), TLR7 and TLR8 recognize single-stranded RNA (ssRNA) found during viral replication, and TLR9 detects unmethylated deoxycytidyl-phosphate-deoxyguanosine (CpG) DNA motifs commonly found in bacterial and viral genomes. Studies have shown that guanosine is also a TLR7 agonist, and binding of guanosine and ssRNA to two distinct sites on TLR7 leads to synergistic activation of TLR7 [20, 21]. Under homeostatic conditions, endogenous nucleic acids are usually not recognized by these endosomal TLRs, but increasing evidences have shown that TLR activation by endogenous RNA or DNA is often associated with the development of autoimmunity and inflammatory diseases [22]. The ligands for TLR10 or TLR11 remain unclear. Evidences have suggested that TLR10 can form a heterodimer with TLR1, TLR2, or TLR6. TLR11 may also play an important role in host defense against certain infection as mice lacking TLR11 were highly susceptible to uropathogenic bacterial infection in the kidney. However, TLR11 may not be functional in human due to the presence of stop codons in the open reading frame of human TLR11 DNA which may represent a form of genetic polymorphism and may lead to failure of the translation of a full-length TLR11 protein [15, 23].

### 2.2. Signaling Pathway of TLRs

Activation of TLR signaling requires homodimerization or heterodimerization of TLRs or with coreceptors, which form an “m”-shaped dimer sandwiching the ligand molecule structure to facilitate dimerization of the intracellular TIR domains and to trigger a downstream signaling cascade [24]. TLR2 is known to form heterodimers with TLR1 or TLR6 to recognize distinct peptidoglycan (PGN) or lipopeptides from gram-positive bacteria or mycoplasma. For example, TLR2-TLR1 recognize the bacterial lipopeptide Pam3CSK4 (tripalmitoyl-S-glycero-Cys-(Lys)4), whereas TLR2-TLR6 recognize bacterial PGN, lipoteichoic acid (LTA), and diacylated lipopeptides such as Malp2 (macrophage-activating lipopeptide-2) [25, 26] (Figure 1). The presence of coreceptors can promote the ligand binding efficiency for several TLRs, such as CD14 for TLR2, TLR4, TLR3, TLR7, and TLR9, CD36 for TLR2 and TLR6, and CD44 for TLR4 [15, 27]. MD2 is a receptor component associated with TLR4 and enables TLR4 to respond to LPS or lipid A [16].

As shown in Figure 1, following ligand-induced dimerization of the ectodomains of TLRs, the intracellular TIR domains of TLRs dimerize and recruit TIR domain-containing adapter proteins, such as MyD88, TIRAP (TIR domain-containing adaptor protein), TRIF, and TRAM (TRIF-related adaptor molecule). Depending on the adapter usage, TLR signaling is generally divided into the MyD88-dependent and TRIF-dependent pathways. All TLRs, except TLR3, use the MyD88-dependent pathway to initiate signaling, and TLR4 uniquely utilizes both MyD88 and TRIF pathways. After TLR engagement, TIRAP mediates recruitment of MyD88, which then forms a complex with IRAK (IL1R-associated kinase) family kinases, including IRAK1, IRAK2, IRAK4, and IRAK-M, to induce TRAF6 (TNFR-associated factor 6) activation. TRAF6, as an E3 ubiquitin ligase, activates TAK1 (transforming growth factor beta-activated kinase 1) through the cooperation with TAB1/2/3 (TAK1-binding protein). Activated TAK1 then phosphorylates the IKK complex (inhibitor of nuclear factor kappa-B kinase), which promotes the degradation of I*κ*B (inhibitor of NF*κ*B), and the dissociated NF*κ*B then translocates to the nucleus for the induction of targeted genes. On the other hand, TAK1 can activate MAPK (mitogen-activated protein kinase) family kinases, including stress-activated protein kinase p38, Jun N-terminal kinase JNK, and signal-regulated kinase ERK1/2; activation of these MAPKs leads to the activation of the heterodimer of ATF2 and c-Jun, called AP-1. AP-1 translocates to the nucleus where it coordinates with NF*κ*B to initiate transcription of various inflammatory cytokines, chemokines, and costimulatory factors [15].

Activation of TLR3 by dsRNA or TLR4 by LPS mediates type 1 interferon (IFN) production via the TRIF-dependent pathway. TRIF is first recruited to the TIR domain of TLRs by TRAM, and TRIF recruits TRAF6 and/or TRAF3. TRAF6 recruits RIP1 (receptor-interacting protein 1) kinase, which activates TAK1 and the subsequent NF*κ*B and MAPK pathways. In contrast, TRAF3 recruits TBK1 (TANK-binding kinase 1) and IKK*ε* (inhibitor of *κ*B kinase *ε*), which in turn lead to the phosphorylation and nuclear translocation of IRF3, an important transcription factor regulating IFN*β* production [28]. Ligand binding of TLR3 also activates the AKT in a TBK1-dependent manner, and AKT contributes to IRF3 phosphorylation by interacting with TBK1 [29, 30]. In contrast, IFN*α* production upon activation of TLR7/8 by ssRNA or TLR9 by CpG-DNA in plasmacytoid dendritic cells (pDCs) is mediated by the MyD88 pathway [31]. A signaling cascade involving MyD88, IRAKs, TRAF6, and IRF7 leads to the activation and nuclear translocation of NF*κ*B and IRF7, which mediate the transcription of IFN*α* in activated pDCs.

## 3. TLRs in Skin Inflammatory Diseases

TLR signaling plays an essential role in host defense against danger signals by producing a diverse range of cytokines, chemokines, antimicrobial peptides, and costimulatory factors, and it is also required for adaptive immunity activation for long-term protection. However, aberrant activation of TLRs may disturb the homeostatic balance of the immune system and may trigger the development of systemic autoimmune diseases. For example, type 1 interferons, which are the key antiviral cytokines induced during viral infection, are potential triggers of several autoimmune diseases such as systemic lupus erythematosus (SLE), psoriasis, rheumatoid arthritis, diabetes mellitus, Sjogren's syndrome, dermatomyositis (DM), and systemic sclerosis [31]. In addition to psoriasis and SLE, unbalanced activation of TLRs may lead to other skin diseases, such as atopic dermatitis, impaired wound closure, diabetic foot ulcers, and skin cancer. Progression of these localized skin diseases may lead to systemic diseases, posing a serious threat to human health and life [12, 32].

### 3.1. Expression and Function of TLRs in Skin Cells

TLRs are expressed by various skin cell types in a cell-specific manner [11, 13]. Keratinocytes, localized at the surface of the skin, is the major epidermal cell type and are the first responders to external pathogens or injury. TLR2 and TLR3 are the most studied TLRs in keratinocytes, whereas the expression levels of other TLRs, such as TLR4, TLR7, TLR8, and TLR9, are much higher in myeloid immune cells compared to keratinocytes [22, 33–39]. Bacterial lipopeptide-mediated TLR2 activation in keratinocytes not only triggers the production of proinflammatory cytokines such as TNF*α* and IL6 but also enhances the tight junction barrier function of the epidermis upon pathogen invasion [37, 39]. In contrast, dsRNA-mediated TLR3 activation is required for normal inflammatory response during viral infection, skin injury, or UV irradiation [33–35]. TLR3 is also required for normal skin barrier repair following tissue damage, and activation of TLR3 induces the expression and function of tight junction components and markedly enhances reepithelialization, granulation, and neovascularization required for wound healing [36, 40].

While the innate immune function of keratinocytes has been extensively studied, the immune functions of dFBs and adipocytes in host defense and tissue repair have only been unrevealed and recognized recently [9, 22, 41, 42]. dFBs express functional TLR2 and TLR4 [43, 44]. TLR2-mediated recognition of bacterial lipopeptides or fungal pathogen *Candida albicans* and TLR4-mediated recognition of LPS stimulate the production of proinflammatory cytokines such as IL6 to promote both innate and adaptive immunity against pathogen invasion [43, 44]. However, excessive activation of TLR2 or TLR4 in dFBs by endogenous DAMPs such as TLR4 ligands hyaluronan, fibrinogen, and other ECM proteins or TLR2 ligand serum amyloid A (SAA) may lead to the pathogenesis of fibrotic skin disorders, such as hypertrophic scarring and systemic sclerosis (SSc) [43–45]. dFBs have the potential to commit to preadipocytes (pAds) which can differentiate into adipocytes upon stimulation. Our group has shown that dermal infection with the gram-positive bacteria *Staphylococcus aureus* (*S. aureus*) triggers a dermal reactive adipogenesis response, characterized by dFB commitment to pAd in response to infection, and then pAd proliferation followed by adipocyte differentiation, and during this process, the antimicrobial peptide cathelicidin (CAMP) is abundantly secreted by differentiating adipocytes, conferring host resistance to the invasive *S. aureus* infection [9, 41, 42]. In vitro, *S. aureus* or TLR2 ligand treatments enhance the adipogenic potential of primary mouse dFBs [42], suggesting that TLR2 activation may drive the commitment of dFB to adipocyte lineage and enable dFB's ability to produce antimicrobial peptide during the subsequent differentiation step. Together, with emerging roles for dFBs in host defense being unrevealed, studies are urgently needed to define the role of TLRs in activating dFBs.

Dendritic cells (DCs), known as the professional antigen-presenting cells (APCs), function as immune sentinels and play a pivotal role in bridging innate and adaptive immunity in the skin [46]. DCs uptake and process antigens and become functional mature antigen-presenting cells followed by migration to lymph nodes, where they prime T cell differentiation and activation to induce adaptive immune responses to microbials, vaccines, and self-antigens. TLRs are critical molecules for antigen presentation and induction of cytokines, chemokines, and costimulated molecules in DCs. Langerhans cells (LCs), a unique subset of APCs located in the epidermis between keratinocytes, rapidly sense PAMPs, DAMPs, or antigens and migrate to lymph nodes to prime T cells to elicit appropriate cutaneous immune responses for host defense [47, 48]. TLR2 is the most prominent TLR expressed in LCs, and LCs also express TLR8, TLR4, and TLR3 [49]. Activation of TLR2 or TLR7/8 in LCs leads to the production of proinflammatory cytokines such as IL12, CCL3, and IL8, whereas TLR3 stimulation in LCs induces the expression of chemokines (CXCL9, CXCL11, and CXCL10) and IFN*β* [47]. DCs in the dermis can be subdivided into conventional DCs (cDCs) and plasmacytoid DCs (pDCs) [50]. While cDCs normally reside in the skin under homeostatic condition, pDCs are not present in healthy skin but rapidly infiltrate the skin dermis upon injury [50, 51]. We and others have shown that cDCs express most TLR family genes at moderate levels whereas TLR7 and TLR9 and their downstream signaling molecule IRF7 are preferentially expressed at high levels in pDCs [22, 52]. This unique TLR expression signature enables pDCs to rapidly respond to ssRNA or DNA and produce high levels of type 1 IFNs, especially IFN*α* family genes, to promote autoimmune activation [22].

**Figure 2:**
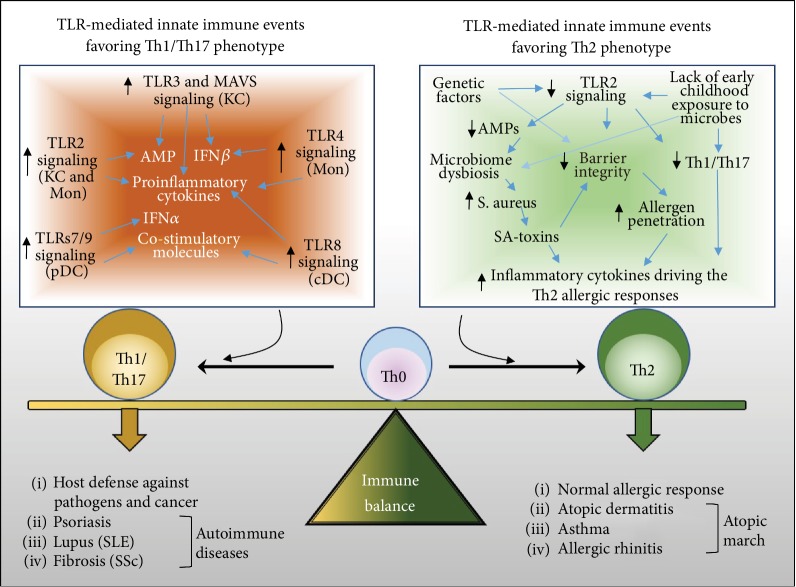
Proposed model for the role of TLR-mediated innate immune events in regulating the Th1/Th17 and Th2 immune balance. The development of Th1/Th17 T cells can be initiated upon innate immune activation of several TLRs, including TLR3 and MAVS (mitochondrial antiviral signaling protein) in keratinocytes (KCs), TLR7 and TLR9 in pDCs, TLR8 in cDCs, TLR2 in KCs and monocytes (Mon), and TLR4 in monocytes. Activation of these TLR-mediated signaling events leads to elevated expression of proinflammatory cytokines, type 1 interferons (including IFN*β* from KCs and IFN*α* from pDCs), antimicrobial peptides (AMP), and costimulatory molecules (on cDCs and pDCs), which ultimately promote the differentiation of T cells from the Th0 to Th1/Th17 phenotype. In contrast, impaired TLR2 may play a role in the development of Th2 immune response. Genetic factors (such as TLR2 polymorphisms) or lack of early childhood exposure to microbes impairs TLR2 expression, and the resultant defective TLR2 signaling leads to decreased expression of antimicrobial peptides (AMPs), compromised epithelial barrier integrity, and decreased expression of Th1/Th17 cytokines. Impaired barrier integrity plays a central role in driving the allergic Th2 immune response by allowing allergens to penetrate through the skin surface. In addition, lack of AMP expression in the skin epidermis promotes dysbiosis of the skin microbiome and overgrowth of *S. aureus*, which releases several virulent toxins that exacerbate the disruption of barrier integrity and the expression of inflammatory Th2 cytokines. Activation of the Th1/Th17 immune system is necessary to promote autoimmunity and host defense against pathogens and cancer cells, but overstimulation of the Th1/Th17 pathway drives the development of several autoimmune diseases, including psoriasis, systemic lupus erythematosus (SLE), and fibrotic skin diseases (e.g., hypertrophic scarring and systemic sclerosis (SSc)). On the other hand, activation of the Th2 immune system is necessary to elicit normal allergic immune responses to allergens or pathogens, but overstimulation of Th2 immune response early in life initiates the progression of allergic diseases including atopic dermatitis, asthma, and allergic rhinitis, a pathological process known as “atopic march.”

TLR-mediated innate immune activation of skin-resident keratinocytes, fibroblasts, and dendritic cells promotes the activation or recruitment of myeloid-derived immune cells such as neutrophils and macrophages or adaptive immune cells such as T cells, leading to immediate and long-term immunity against danger signal. During wound healing, cessation of the initial defensive/inflammatory phase is required for the subsequent proliferative and remodeling phases to complete the healing process and return to homeostatic condition. Therefore, unresolved or excessive inflammation not only can lead to the development of autoimmune skin diseases such as psoriasis, atopic dermatitis, rosacea, lupus, and systemic sclerosis but also can lead to defective or aberrant wound healing as seen in wound ulcers, diabetic foot ulcers, keloid, or hypertrophic scars [12, 53, 54] (Figure 2). We will next focus on reviewing the roles of TLRs in the pathogenesis of the two most common inflammatory skin diseases: psoriasis and atopic dermatitis.

### 3.2. Innate Immune Activation of TLRs and Psoriasis Initiation

Psoriasis is a chronic, recurrent, genetic autoimmune skin disorder featured by well-demarcated, raised areas of erythematous plaques, often covered by silvery scaling [55]. It is estimated that ~1.7% of the world population is affected by psoriasis, including ~3% of the US and European populations and ~0.5% of the Chinese or Asians [56]. Principal histological features of psoriasis are hyperplastic epidermis, increased vascularity in the dermis, and dermal infiltration with inflammatory leukocytes. There is no cure for psoriasis, and the recurrence of psoriasis can be triggered by several factors such as skin injury, infection, stress, and drugs such as *β* blockers, lithium, type 1 interferons, and imiquimod [57].

Psoriasis is considered a T cell-mediated disease, because T cell-derived cytokines such as IL17A and IL22 are responsible for the hyperproliferation and aberrant differentiation of keratinocytes that ultimately leads to psoriatic plaque formation. However, PRR-mediated recognitions of DAMPs or PAMPs and the resultant innate immune responses in keratinocytes or pDCs are believed to be the early initiating events in psoriasis that drive the subsequent adaptive immunity and autoimmunity development. Upon DAMP or PAMP stimulation, keratinocytes are capable of producing an array of proinflammatory cytokines, such as IFN*β*, IL1*β*, IL36, TNF, IL6, IL8, IL25, and CXCL10, to initiate the inflammatory T cell phenotype in psoriasis [22, 58–60].

Skin injury even superficial tattoos can trigger psoriasis, and this is known as the “Koebner phenomenon.” We have recently shown that PRR-mediated activation of the innate immune responses in keratinocytes plays a role in triggering psoriasis upon skin injury [22]. During skin injury, damaged cells release DAMPs such as dsRNA, ssRNA, and DNA, and we have found that the antimicrobial peptide LL37 which is upregulated during wounding enables dsRNA recognition in keratinocytes through the TLR3 and mitochondrial MAVS (mitochondrial antiviral signaling protein) signaling pathway, leading to IFN*β* production from KC or pDC, respectively [22, 61]. Dr. Gilliet's group has also shown that LL37 can also enable ssRNA or DNA recognition by TLR7 or TLR9 in pDCs, which then produce a large quantity of IFN*α* [51, 61]. The self-ssRNA-LL37 complexes also activate cDC through TLR8, leading to the production of TNF*α* and IL6 and cDC maturation [62]. Direct comparison of the transcript levels of PRRs in KC, pDC, and cDC reveals that while TLR3 is expressed at similar levels in all cells, MAVS is preferentially in KCs, TLR4 and TLR8 are expressed at higher levels in cDCs, and TLR7-TLR9 are preferentially expressed by pDCs [22]. These cell type-specific expression patterns of PRRs can explain the cell type-dependent responsiveness to various DAMPs or PAMPs during skin injury. Type 1 IFNs, including IFN*β* from KCs and IFN*α* from pDCs, serve as early cytokines released upon injury to promote cDC activation and maturation with consequent Th17 T cell development and the beginning of the autoimmune self-amplification loop that drives pathogenic hyperproliferation of KCs and manifestations of psoriasis.

The roles of TLR2 or TLR4 in psoriasis still remain unclear. The expression of TLR2 and TLR4 on peripheral blood mononuclear cells and keratinocytes is elevated in patients with psoriasis [63, 64]. There is also an association between polymorphisms within TLR4 with chronic plaque type psoriasis and psoriatic arthritis [65]. A recent study has shown that epidermal infiltration of neutrophils drives inflammatory responses in the skin through activation of the epidermal TLR4-IL36R crosstalk in the imiquimod- (IMQ-) induced psoriasis-like mouse model [66]. Additionally, heat shock proteins (HSPs), such as HSP27, HSP60, HSP70, and HSP90, are overexpressed in KCs of psoriasis patients, and these HSPs can function as autoantigens to activate antigen-presenting cells (APC) through TLR4 to promote APC maturation and secretion of TNF*α* and IL12 [67–69].

In summary, psoriasis is a complicated autoimmune disease mediated by the dynamic interplay between the innate and the adaptive immune cells. TLR-mediated activation of keratinocytes, pDCs, and/or cDC initiates early innate immune events that link to T cell activation and the development of autoimmunity in psoriasis. Current psoriasis therapies targeting T cell activation are effective in clinical trials [57], but potential problems including lack of long-term efficacy and rapid relapse of the disease upon drug removal [70–72] suggest that targeting the T cell alone is not enough. Targeting PRR-mediated innate immune activation of KCs or pDCs in the combination of T cell therapies may result in more sustainable effect to treat psoriasis.

### 3.3. Dysbiosis of Skin Microbiome, Impaired TLR2 Function, and Atopic Dermatitis

Atopic dermatitis (AD), a chronic, inflammatory skin disease characterized by an eczema-like lesion and intense pruritus and high serum immunoglobulin E (IgE), is a major health problem worldwide affecting 15~20% of children and 2~3% of adults [73–76]. AD often begins early in infancy around 3 months of age, and about 80% children have a spontaneous remission of the disease before adolescence, whereas the remaining 20% continue to have eczema into adulthood. Children with persistent AD symptoms often develop asthma and/or allergic rhinitis from 3 years of age, a process known as “atopic march” [75, 77]. Studies suggest that environmental factors may be critical in disease progression of AD. First, the prevalence of symptoms of AD is about 5~10 times higher in developed countries such as the United Kingdom, Japan, Australia, and the USA compared to developing countries such as Iran and China [75, 76]. Furthermore, the development of AD is inversely associated with early childhood exposure to infections or microbe-rich environment such as living with older siblings or pets or on a farm [78] (Figure 2). A hygiene hypothesis has therefore been proposed to describe the protective influence of microbial exposure to early life on the development of AD [78].

Recent studies have shown that dysbiosis of skin microbial community (microbiome) may promote disease progression of AD (Figure 2). The lesional skin of AD patients is often colonized with *S. aureus*, and skin *S. aureus* colonization not only positively correlates with disease severity but also precedes the clinical diagnosis of AD, suggesting that *S. aureus* may actively contribute to AD pathogenesis [79–81]. High-throughput DNA sequencing of the bacterial 16S rRNA has revealed that while bacterial composition is highly diverse on healthy skin, there is a dramatic loss of skin microbial diversity during AD flares, and the proportion of *Staphylococcus* shifts from ~20% in normal skin to a dominant ~90% in AD flare [82]. The main consequence of increased colonization of *S. aureus* in AD skin is the exacerbation of the allergic Th2 inflammatory response by staphylococcal enterotoxins (also known as “superantigens”) and phenol-soluble modulins (PSMs) [83–85] and the disruption of epidermal barrier integrity mediated by other virulence factors of *S. aureus* (e.g., *S. aureus* proteases, such as aureolysin and V8 protease) [86]. A recent study from the Gallo lab has also shown that *S. aureus*-derived PSM*α* also induces the expression of endogenous protease activity in keratinocytes, further contributing to the disruption of barrier homeostasis [87]. Lack of early childhood exposure to beneficial microbes likely promotes dysbiosis of the skin microbiome. Indeed, studies from the Gallo group have shown that the commensal bacteria *S. epidermidis* can secrete antimicrobial peptide or DNA analog to suppress the growth of pathogenic bacteria *S. aureus* or *group A Streptococcus* (GAS), and furthermore, the commensal bacteria *S. hominis* can suppress toxin production from *S. aureus* through an autoinducing peptide [87–89]. Together, these evidences suggest that imbalanced skin microbiome composition and overgrowth of *S. aureus* are key triggering factors for the pathogenesis of atopic dermatitis.

Impaired TLR2 function has been associated with the pathogenesis of atopic dermatitis (AD) (Figure 2). Genetic polymorphisms of TLR2 have been identified to be associated with AD [90, 91], and TLR2 was also found to be downregulated in macrophages or peripheral blood mononuclear cells (PBMC) isolated from peripheral blood from AD patients [92–94]. Additionally, macrophages or PBMC from AD patients treated with TLR2 ligands produce significantly less TH1/TH17 cytokines such as interleukin 6 (IL6), IL1*β*, IFN*γ*, IL12, and IL17F and IL22, but more TH2 cytokine IL5 [93, 95]. *S. aureus*-mediated TLR2 activation is also strongly impaired in Langerhans cells from AD skin [48]. Confocal microscopy of skin sections from normal or AD patients revealed that TLR2 is normally expressed throughout the epidermis but limited to the basal keratinocytes in AD skin [92]. In normal keratinocytes, activation of TLR2 rapidly increases the expression of tight junction (TJ) protein claudin1 and antimicrobial peptide (AMP) genes such as *β*-defensins and cathelicidin in differentiated epidermal layers [96]. However, the lesional skin of AD patients expresses significantly decreased levels of TJ proteins as well as AMPs [96, 97], indicating that TLR2 signaling is impaired in the suprabasal layers of the epidermis where these genes are expressed. Therefore, impaired TLR2 signaling in various skin cells from AD patients may ultimately skew the immune response to *S. aureus* toward a TH2-dominant immune phenotype, a hallmark of allergic diseases such as AD. Cytokines produced by TH2 lymphocytes including IL4, IL5, and IL13 are central to the pathogenesis of atopic diseases [98].

While TLR2 signaling is impaired during the acute phase of AD, it has also been suggested that aberrant activation of TLR2 may play a role in promoting the development of the Th1 immune pathway that leads to the exacerbation and persistence of inflammation during the chronic phase of AD [99, 100]. Thymic stromal lymphopoietin (TSLP), a cytokine highly expressed by epidermal keratinocytes in AD skin, has been recognized as the master regulator linking innate response at the barrier surface to TH2-skewed adaptive immune response in atopic diseases [101, 102]. The expression of TSLP can be triggered by exposure to environmental factors, such as allergens and microorganisms, and elevated TSLP expression is observed before the development of clinical AD phenotypes in both human and mice [101, 103], suggesting that TSLP is the early initiating factor driving AD pathogenesis. In vitro, TLR ligands (including TLR3 ligand poly (I:C), TLR2-6 ligand FSL1, and TLR5 ligand flagellin) or isolated *S. aureus* membrane components induce TSLP expression and release from primary human keratinocytes, and TSLP expression can also be regulated by vitamin D3 and TH2 cytokines (IL4 and IL13) in human KCs [104]. Considering that TSLP can be induced upon activation of several TLRs (including TLR2 and TLR3) or by TLR-independent mechanisms [101], it is still unclear whether aberrant activation of TLR2 contributes to high TSLP expression in AD. Future studies are needed to define the role of TLR2 in TSLP expression and in converting AD from a Th2-dominant acute phase to a Th2-Th1 mixed chronic inflammation phase.

## 4. TLR-Targeted Therapies

TLRs play important roles in linking innate and adaptive immune responses to initiate immediate as well as long-term host defense against danger signals, and dysregulations of TLRs are responsible for the pathogenesis of several inflammatory skin diseases, and therefore, targeting TLRs is of great therapeutic potential to treat skin diseases. Several TLR agonists or antagonists or TLR modulators have been approved or are currently in development to treat skin diseases [105]. We will next review TLR's therapeutic implication, recent advances, and future prospects in treating skin diseases.

### 4.1. Therapeutic Use of TLR Ligands to Boost Host Immunity against Pathogens or Cancer

TLR agonists have been used to treat infectious skin diseases by boosting host innate immune defense against pathogens. *Candida albicans*, a fungal member of the normal human skin microbiome, is normally harmless, but in immunodeficient patients, it can cause life-threatening infections. Amphotericin B (AmB), a commonly used antifungal agent, stimulates several TLRs (TLR1, TLR2, and TLR4) followed by the production of proinflammatory cytokines such IL6, IL8, and TNF, boosting the host's immunity against *C. albicans* [106]. Caspofungin (echinocandins), a new class of antifungal drugs, inhibits the synthesis of *β*-glucan in the fungal cell wall by influencing the interactions between Dectin1 and TLR2, TLR4, or TLR9 [107]. TLR ligands have also been used for the treatment or vaccine development for herpes simplex virus (HSV) [105]. In mice, HSV vaccines adjuvanted with the TLR9 agonist unmethylated CpG are superior to the unadjuvanted vaccine at eliciting a robust HSV-specific cell-mediated immune response [108].

TLR agonists have also been used to boost locoregional and systemic immunity against cancer. Imiquimod (IMQ), a TLR7/8 ligand, is the first US FDA-approved drug to treat external genital and perianal warts and then approved for actinic keratosis and basal cell carcinoma (BCC), the most common skin cancer worldwide [109]. The effect of IMQ is mediated by recruitment and activation of pDC, cDC, or macrophages through TLR7/TLR8, leading to the production of cytokines including type 1 IFNs, IL1, IL6, and TNF followed by the development of cell-mediated adaptive immunity against cancer cells [109]. Due to its autoimmune-stimulatory capacity, a known side effect of IMQ is the development of psoriasis-like skin inflammation in both human and mice, and therefore, topical application of IMQ has been commonly used as a method to trigger psoriasis-like skin inflammation in mice. Synthetic unmethylated CpG type B oligodeoxynucleotide CpG 7909, the TLR9 agonist that stimulates DC, macrophages, or NK cells, has been shown to be effective against BCC and metastatic melanoma [110, 111]. Other TLR ligands, such as TLR3 ligand poly (I:C), a synthetic analog of viral dsRNA, can be used in combination with antitumor nanoparticles to promote melanoma regression in mice by promoting melanocyte apoptosis and shifting macrophages to a proinflammatory and tumoricidal phenotype [112].

### 4.2. Therapeutic Effects of TLR Inhibition in Psoriasis

While the TLR7-8 agonist imiquimod triggers psoriasis, synthetic oligonucleotides, antagonists for TLR7-9, can suppress Th1 and Th17 immune development in a mouse model of IL23-induced psoriasis [113]. In addition, several oligonucleotide-based antagonists of TLR7-9 such as IMO-3100 and IMO-8400 have been shown to be safe and effective in phase 2 clinical trials in patients with moderate-to-severe plaque psoriasis by blocking the activation of the IL17 pathway [114].

Conventional psoriasis therapies, including topical applications of vitamin D analogs or vitamin A analogs, have also been shown to exert their anti-inflammatory effects by modulating TLR function. Vitamin D3 downregulates the expression of TLR2, TLR4, and TLR9 and suppresses TLR9-mediated cytokine production in human monocytes [115], and the vitamin D analog calcipotriol attenuates CpG-mediated elevation of TLR9 and MyD88 expression in pDCs [116]. Retinoids, namely, vitamin A and its metabolites, have been used to treat psoriasis since the 1980s. Retinoid-mediated activation of retinoic acid receptors (RAR) and retinoid X receptors (RXR) improves the symptoms of psoriasis by regulating cell proliferation/differentiation as well as by suppressing inflammation [117]. Retinoid analog can reduce the expression of TLR2 and its coreceptor CD14 in human monocytes and therefore prevent TLR2-mediated innate immune response to microbes [118, 119].

Together, inhibition of TLRs by specific TLR antagonists or by natural compounds such as vitamin A or D analogs attenuates the activation of the innate immune system that initiates the autoimmune cascade in psoriasis. Although new biological drugs targeting T cell activation molecules such as TNF*α* (such as etanercept, adalimumab, and infliximab), IL12 and IL23 (such as ustekinumab), IL23 (such as guselkumab, tildrakizumab, and risankizumab), IL17A (such as secukinumab and ixekizumab), or IL17 receptor A (such as brodalumab) have shown to be safe and efficacious in recent psoriasis clinical trials, however, lack of long-term efficacy and rapid regain of psoriasis upon removal of these drugs suggest that preventing adaptive immune activation alone is not sufficient to treat psoriasis. Targeting TLRs or PRRs in combination with T cell therapy may result in more sustainable effect to treat psoriasis.

### 4.3. TLRs and Atopic Dermatitis

As we have described earlier, impaired TLR2 function plays a role in driving loss of barrier integrity and the immune system imbalance (Th2 dominance) during the acute phase of AD, but aberrant activation of TLR2 may lead to Th1 immune development during the chronic phase of AD and may also lead to the production of keratinocyte-specific cytokine TSLP that drives the allergic immune responses. Therefore, strategies that finely modulate TLR2 expression or function hold promise in restoring barrier function and immune balance in AD.

Topical calcineurin inhibitors (TCIs), including tacrolimus and pimecrolimus, are FDA-approved drugs for the treatment of AD. TCIs block the activity of the enzyme calcineurin, to prevent the activation of the nuclear factor of activated T cells (NFAT), which in turn blocks cytokine IL2 production as well as the subsequent T cell activation and proliferation [120–122]. It has also been reported that the abnormal expression of TLR1 and TLR2 can be normalized after a 3-week treatment with tacrolimus ointment [123], suggesting that TCIs may exert their therapeutic effects by restoring normal function of TLR2 signaling in AD.

## 5. Conclusion

Skin, located at the first line of defense, is constantly exposed to pathogenic or danger factors from the environment. TLRs, the key pattern recognition receptors, are involved in the recognition of PAMPs or DAMPs, initiation of innate immune responses, regulation of adaptive immune responses, and ultimately development of immediate and long-term immunity against pathogens. There is a growing body of evidence demonstrating that TLRs play indispensable roles in the pathogenesis of several inflammatory skin diseases, and therefore, therapeutic strategies have been developed and studied to target TLRs to either boost immunity against pathogens or cease aberrant activation of TLRs that drives autoimmune activation. But with recent success in the new biological drugs targeting T cells, the effector cell type at the downstream of disease progression, therapeutic approaches targeting innate immune activation during early stages of disease progression become less favorable. However, inhibiting the activation of the adaptive immune activation alone, without blocking the early innate immune events, can only alleviate disease symptoms but cannot cure the disease and may lead to rapid regain of inflammation upon drug removal. Future studies will be needed to develop targeted therapies for TLRs or PRRs which may be used in combination with T cell-targeted therapy to achieve more sustainable interventions to treat inflammatory skin diseases, such as psoriasis or atopic dermatitis.
